# The contributions of cancer cell metabolism to metastasis

**DOI:** 10.1242/dmm.032920

**Published:** 2018-05-03

**Authors:** Gloria Pascual, Diana Domínguez, Salvador Aznar Benitah

**Affiliations:** 1 Institute for Research in Biomedicine (IRB Barcelona), Oncology Department, The Barcelona Institute of Science and Technology (BIST), 08028, Barcelona, Spain; 2 Catalan Institution for Research and Advanced Studies (ICREA), 08010, Barcelona, Spain

**Keywords:** Cancer, Metabolism, Metastasis, Epigenetics

## Abstract

Metastasis remains the leading cause of cancer-related deaths worldwide, and our inability to identify the tumour cells that colonize distant sites hampers the development of effective anti-metastatic therapies. However, with recent research advances we are beginning to distinguish metastasis-initiating cells from their non-metastatic counterparts. Importantly, advances in genome sequencing indicate that the acquisition of metastatic competency does not involve the progressive accumulation of driver mutations; moreover, in the early stages of tumorigenesis, cancer cells harbour combinations of driver mutations that endow them with metastatic competency. Novel findings highlight that cells can disseminate to distant sites early during primary tumour growth, remaining dormant and untreatable for long periods before metastasizing. Thus, metastatic cells must require local and systemic influences to generate metastases. This hypothesis suggests that factors derived from our lifestyle, such as our diet, exert a strong influence on tumour progression, and that such factors could be modulated if understood. Here, we summarize the recent findings on how specific metabolic cues modulate the behaviour of metastatic cells and how they influence the genome and epigenome of metastatic cells. We also discuss how crosstalk between metabolism and the epigenome can be harnessed to develop new anti-metastatic therapies.

## Abstract (precis)

**Summary:** In this Review the authors summarize recent findings on how specific metabolic cues modulate and influence the behaviour, genome and epigenome of metastatic cancer cells. They also discuss how the crosstalk between metabolism and the epigenome of metastatic cells can be harnessed to develop new anti-metastatic therapies.

## Introduction

Metastasis is the leading cause of cancer-related deaths worldwide yet, at the cellular level, it is an inefficient process – only a small fraction of cells shed from a primary tumour into the bloodstream or lymphatic system will successfully complete all the sequential steps of the metastatic cascade (Oskarsson et al., 2014). The mechanisms by which some tumour cells detach from the primary lesion to colonize distant sites are beginning to be deciphered, providing new avenues for therapeutic intervention. For instance, pro-metastatic events common to most solid tumours include the reversible transition of tumour cells from an epithelial to a mesenchymal state [epithelial-to-mesenchymal transition (EMT); see Box 1 for a glossary of terms], as well as interactions with tumour-activated stromal cells, such as pericytes, fibroblasts, endothelial cells, adipocytes or immune cells (Calon et al., 2012, 2015; Cao et al., 2014; Chaffer and Weinberg, 2011; Chen et al., 2011; Goel and Mercurio, 2013; Kalluri and Zeisberg, 2006; Lu et al., 2011; McAllister and Weinberg, 2014; Nieman et al., 2011; Obenauf et al., 2015; Oskarsson et al., 2011, 2014; Paolino et al., 2014; Peinado et al., 2012; Sevenich et al., 2014; Ugel et al., 2015; Valiente et al., 2014; Wculek and Malanchi, 2015; Zhang et al., 2013). Tumours also secrete metastasis-promoting exosomes that contain various proteins, mRNAs and microRNAs, to establish a distant pro-metastatic niche (Costa-Silva et al., 2015; Ghajar et al., 2013; Obenauf et al., 2015; Peinado et al., 2012; Zhou et al., 2014; Zomer et al., 2015).

**Box 1. Glossary**

**Anaplerosis:** the replenishment of TCA cycle intermediates that have been used for biosynthetic processes.

**Dormant/quiescent cancer cells:** cells that remain in a state of dormancy or quiescence in response to intrinsic or extrinsic stimuli that arrest mitosis and cell growth.

**Driver mutation:** mutational events that confer cancer cells with either growth or survival advantages and therefore favour (drive) tumour development.

**Enhancers:** short DNA regions that regulate gene expression by recruiting transcription factors that establish and maintain cell-specific transcriptional signatures. They can be located far away from the genes they regulate.

**Epithelial-to-mesenchymal transition (EMT):** a process by which epithelial cells gain mesenchymal cell properties, including migratory and invasive traits. EMT occurs during embryogenesis, wound healing and during the malignant transformation of cancer cells.

**Ketogenic diets:** low-carbohydrate and high-fat diets that force the body to preferentially mobilize stored lipids for energy production, resulting in increased levels of ketone compounds.

**Metformin:** a drug commonly used to treat type-2 diabetes that inhibits OXPHOS and consequently reduces ATP production in mitochondria, favouring cellular ATP production via glycolysis.

**Omental fat pad:** an area of fat tissue in the abdominal cavity that surrounds the intestines.

**Orthotopic model:** an animal model, most commonly mouse, in which human tumour cells are injected or implanted into the equivalent organ or tissue that the human cancer originated from.

**Oxidative phosphorylation (OXPHOS):** this process is the most efficient way to produce ATP in eukaryotic cells via the electron flow that occurs in the mitochondrial inner membrane.

**Pioneer transcription factor:** a transcription factor that can bind compacted chromatin to recruit chromatin remodelling proteins and other transcription factors; these transcription factors are important for determining cell fate.

**Preconditioned niche:** an environment at a distance from the primary tumour that is modified and generates specific signals prior to the arrival of disseminated cancer cells; this environment can facilitate subsequent tumour cell infiltration and colonization.

**Tricarboxylic acid (TCA) cycle:** this metabolic pathway, also known as the Krebs cycle, produces electron carriers for ATP generation through the electron transport chain, which takes place in mitochondria.

**Triple-negative breast cancer (TNBC):** breast carcinoma histologically assessed as negative for estrogen receptors (ER−), progesterone receptors (PR−) and HER2 receptors (HER2−).

**Warburg effect:** process by which tumour cells increase their glucose uptake and convert pyruvate to lactate through aerobic glycolysis under normal conditions of oxygen and glucose availability, yielding ATP, increased NADPH reductive power and metabolic intermediates, instead of coupling glycolysis to the TCA cycle and OXPHOS pathways.

The field of cancer research historically posited that tumour progression entails the progressive accumulation of genetic mutations and the sequential selection of sub-clones – a process that culminates in metastasis as a clinical manifestation of late-stage disease (Merlo et al., 2006; Nowell, 1976). However, recent advances in whole-genome sequencing indicate that, at a very early stage, cancer cells harbour combinations of driver mutations (Box 1) that endow them with metastatic competency (Calon et al., 2012; Goel and Mercurio, 2013; Kalluri and Zeisberg, 2006; Nieman et al., 2011; Zhang et al., 2013). Furthermore, findings from metastatic assays performed *in vivo* suggest that metastatic cells reach distant organs early during primary tumour growth, yet can remain dormant (Box 1), and untreatable, for long periods of up to several years before generating metastases, which are often fatal (Cao et al., 2014; Kalluri and Zeisberg, 2006; Zhang et al., 2013). These studies have revealed that, early during tumorigenesis, the specific driver mutations that confer tumour cells with selective advantages might be the same mutations that provide them with the competency to metastasize (Jacob et al., 2015; Patel and Vanharanta, 2016; Vanharanta and Massagué, 2013). These findings indicate that tumour cells require additional local and systemic influences to metastasize, and imply that our lifestyle could impact tumour progression, which in turn suggests that such lifestyle factors could be modulated if understood. Nevertheless, we are only beginning to understand the nature of the factors that promote metastasis, their origin, and why not all tumour cells respond to them in the same way.

The recent and exciting identification of metastasis-initiating cells (MICs) in different types of tumours allows us to explore what distinguishes metastatic cells from their non-metastatic counterparts (Dieter et al., 2011; Hermann et al., 2007; Lawson et al., 2015; Pascual et al., 2017; Patrawala et al., 2006; Roesch et al., 2010; Wculek and Malanchi, 2015). One particularly interesting aspect of metastatic cells is that they seem to be strongly influenced by specific types of metabolism and their derived metabolites. For instance, lipid metabolism is emerging as an essential factor in tumour progression (Baenke et al., 2013; Pascual et al., 2017). Importantly, intracellular metabolic changes might establish and sustain transcriptional programmes required for metastatic competency, as exemplified by the strong link between specific metabolites and the epigenetic machinery that controls gene expression (Fan et al., 2015; Kinnaird et al., 2016).

In this Review, we discuss recent insights into the metabolic plasticity of cancer cells and the way in which their metabolic processes can contribute to their metastatic transformation. We highlight the emerging role of lipid metabolism as an important source of cancer metabolic heterogeneity, provide an overview of the crosstalk that occurs between metabolic processes and the cancer cell epigenome, and examine how lifestyle influences, such as diet, might affect cancer progression. We also discuss the therapeutic potential of targeting metabolism during cancer progression, highlighting novel and experimental drugs currently under preclinical investigation.

## Metabolic heterogeneity of cancer stem cells

Cancer stem cells (CSCs) sustain the growth of the tumour mass and are responsible for therapy failure and patient relapse (Blanpain, 2013). The identification and characterization of CSCs in a number of malignancies is paving the way towards developing novel CSC-targeted anti-cancer approaches (Collins et al., 2005; Eramo et al., 2008; Hermann et al., 2007; Kreso et al., 2013; Li et al., 2007; Prince et al., 2007; Ricci-Vitiani et al., 2007; Singh et al., 2004; Wu, 2008; Patrawala et al., 2006). One important conclusion of several of these studies is that CSCs display molecular and functional heterogeneity. Interestingly, this heterogeneity seems to be established early during tumorigenesis because genetically distinct CSC sub-clones are already present in primary tumours, some of which fade or become dominant during tumour progression and response to chemotherapy (Ben-David et al., 2017; Shlush et al., 2017; Zehir et al., 2017). However, it is still a matter of debate whether all cells capable of initiating and promoting primary tumour growth are equally competent to initiate metastasis. Substantial evidence suggests that only a few CSC clones present within a primary tumour possess the ability to behave as MICs (Campbell et al., 2010; Roesch et al., 2010; Wculek and Malanchi, 2015; Pascual et al., 2017). As these clones do not harbour new mutations relative to the primary tumour, non-genetic factors are likely to be required to promote their metastatic competency (Hansen et al., 2011). Thus, local and systemic signals might endow certain CSC clones with the ability to colonize distant organs, underlying the functional diversity of a population of genetically identical cancer clones (Kreso et al., 2013).

Intriguingly, the functional heterogeneity of CSCs might require them to use different types of metabolism. As early as 1926, Otto Warburg reported that tumour cells do not generally couple glycolysis to the tricarboxylic acid (TCA) cycle (Box 1) and oxidative phosphorylation (OXPHOS; Box 1), even under normal conditions of oxygen and glucose availability (Warburg, 1925). Instead, they convert pyruvate to lactate through aerobic glycolysis, to yield ATP, NADPH reductive power and metabolic intermediates for cellular biosynthesis (Koppenol et al., 2011; Vander Heiden et al., 2009). The Warburg effect (Box 1), although inefficient, allows for periods of increased biosynthetic demand (Vander Heiden and DeBerardinis, 2017). Nonetheless, cancer cells have functional mitochondria and can use glycolytic or oxidative metabolic programmes to obtain energy when confronted with different scenarios. This is particularly true for CSCs from different types of tumour, which generally and predominantly rely on OXPHOS to cope with their energetic demands, as compared to differentiated tumour cells (Lagadinou et al., 2013; Sancho et al., 2015; Viale et al., 2014) but can revert to glycolysis and enhanced glucose uptake under alternative microenvironments (Dong et al., 2013; Marin-Valencia et al., 2012). For instance, patient-derived CD133^+^ pancreatic CSCs display enhanced mitochondrial respiration and impaired glycolytic plasticity relative to their CD133^−^ non-CSC counterparts, rendering these cells more vulnerable to metformin (Box 1), which inhibits their mitochondrial respiration. Nevertheless, some CSCs within the CSC pool develop metformin resistance through an intermediate metabolic state that involves increased glycolysis and reduced mitochondrial oxygen consumption (Sancho et al., 2015).

Lipids and lipid metabolism have been also linked to CSC function. For instance, ovarian CSCs require *de novo* fatty acid (FA) synthesis and lipid desaturation, through the activity of stearoyl-coA desaturase 1 (SCD1), to promote tumour initiation in a nuclear factor kappaB (NFκB)-dependent manner (Li et al., 2017). In human breast cancer cell lines, expression of the mitochondrial protein lactamase beta (LACTB) is significantly downregulated; LACTB downregulation is required for the synthesis of two phospholipids, phosphatidyl ethanolamine (PE) and LysoPE, that are essential for membrane biosynthesis (Keckesova et al., 2017). Thus, the ability of CSCs to adjust their metabolic state – their metabolic plasticity – likely influences how they contribute to tumour progression and relapse. The following section will address how CSCs modulate their metabolic activity to adapt to environmental cues when confronted with distinct conditions and microenvironments.

## Tumour–microenvironment metabolic crosstalk in metastasis

How cancer cells engage in reciprocal communication with their tumour microenvironment (TME) affects tumour initiation (Oskarsson et al., 2014; Pein and Oskarsson, 2015; Shiozawa et al., 2011). However, we have only recently begun to understand the importance of the interactions between cancer cells and the TME in promoting metastatic initiation and macroscopic metastatic growth.

### Metabolic preconditioning of the metastatic niche

**Fig. 1.**
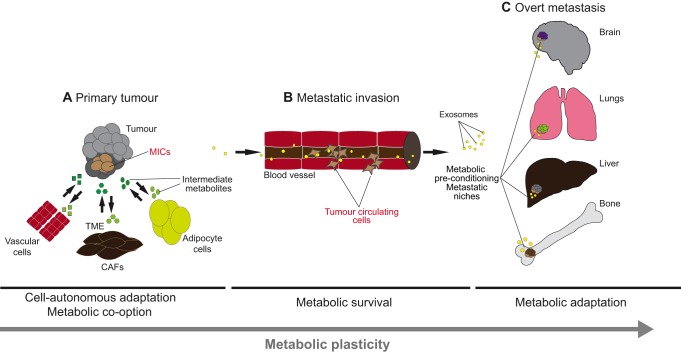
**Cancer metabolic plasticity contributes to metastatic disease.** (A) Genetic mutations and epigenetic alterations in combination establish unique populations of tumour-initiating cells (TICs). Only certain TICs take advantage of the surrounding cells that constitute the tumour microenvironment (TME), such as vascular cells, cancer-associated fibroblasts (CAFs) and adipocytes, as well as their systemic environment, to exchange and hijack metabolites (shown in green) that support TIC survival. TICs hijack metabolites while egressing out of the primary lesion, thereby becoming metastasis-initiating cells (MICs). (B) As metastatic cells reach different distant organs via the vasculature, they adopt unique metabolic states and engage in further metabolic crosstalk with the (C) metastatic niches that form, for example, in the bone, lungs, liver and brain, ultimately supporting their survival. Tumour cells also secrete metastasis-promoting exosomes (yellow) that contain various proteins and RNAs that contribute to establish distant pro-metastatic niches.

An intriguing aspect of the metastatic process is that metastatic niches are preconditioned (Box 1) prior to the arrival of disseminated cancer cells from primary tumour sites (Kaplan et al., 2005; Peinado et al., 2017). Accumulating evidence indicates that specific metabolic changes are associated with the establishment of these niches, although whether these changes are tumour-specific or not remains unclear. For instance, factors secreted by the primary tumour can be transferred to neighbouring or distant cells via exosomes to metabolically modulate the TME and favour metastasis (Peinado et al., 2017) (Fig. 1). Thus, in a human xenograft model, circulating breast-cancer-derived vesicles that contain the microRNA miR-122 have been found to downregulate the expression of glucose transporter 1 (GLUT1) and pyruvate kinase M1/M2 (PKM1/2) isozymes, thereby reducing glucose uptake in lung and brain non-tumour cells. This in turn increases the local pool of glucose available for the incoming metastatic cancer cells (Fong et al., 2015). Similarly, in an *in vivo* experimental model of metastasis in mice, disseminated human colorectal cancer cells were found to secrete the protein creatine kinase brain-type (CKB) in the liver, where it catalyses the conversion of extracellular ATP and hepatic creatine into phosphocreatine, which is incorporated into metastatic cells via the creatine transporter, SLC6A8. Increased phosphocreatine availability in the extracellular space fuels the generation of ATP within metastatic cells, which is essential to meet the intense energetic requirements involved in colonizing the liver (Loo et al., 2015).

### Hypoxia and cancer cell metabolism

The unique unstructured capillary network of an exponentially growing tumour generates areas of low oxygen diffusion and consumption, a phenomenon termed hypoxia. Upon arrival at distant organs, metastatic cells probably lack a proper vasculature to provide them with a plentiful oxygen supply. Importantly, signals triggered by hypoxic conditions are a feature of aggressive tumours, and are associated with poor prognosis and high levels of metastasis (Rankin and Giaccia, 2016; Semenza, 2016).

A key aspect of the hypoxic response is the metabolic adaptation of malignant cells to overcome cell death (Benjamin et al., 2012). The hypoxia-inducible transcription factor 1 alpha (HIF1-α, or HIF1a), is an important transducer of hypoxic signals and drives the expression of GLUTs and glycolytic enzymes, such as hexokinase 2 (HK2), phosphofructokinase 1 (PFK1) and lactate dehydrogenase A (LDHA), while inhibiting the expression of pyruvate dehydrogenase kinase 1 (PDK1). This response reduces the flux of pyruvate into the TCA cycle, thereby decreasing the rate of OXPHOS and oxygen consumption (Semenza, 2010). In certain tumours, hypoxia also alters lipid metabolism by promoting the use of alternative carbon sources, such as acetate, to sustain FA synthesis and tumour growth. This lipid metabolic switch is associated with the transcriptional control of acetyl-CoA synthetase-2 (ACSS2) by HIF signalling, and by *ACSS2* copy-number gains*.* Interestingly, *ACSS2* expression is specifically increased in metastatic cells (Schug et al., 2015). In addition, hypoxic glioblastoma and mammary gland tumours accumulate lipid droplets (LDs) in a HIF1a-dependent manner, through the uptake of FAs via the fatty-acid-binding proteins 3 and 7 (FABP3 and FABP7). Lipid droplets, in turn, sustain cancer cell survival upon re-oxygenation by providing a lipid reservoir that ensures continued ATP production via FA β-oxidation and by protecting cells from reactive oxygen species (ROS) toxicity by reducing NADPH generation (Bensaad et al., 2014). However, whether and how these pathways contribute to the survival of metastatic cells upon their arrival at distant sites is currently unknown.

### Metabolic coupling between tumour cells and stroma promotes cancer spread

One interesting aspect of tumour cells is their ability to shape the metabolism of their TME to ensure a plentiful supply of energy, through a process known as metabolic coupling. Metabolic coupling can occur either between tumour cells or between tumour and stromal cells. For instance, metastatic ovarian cancer cells induce the release of stored lipids from the omental fat pad (Box 1) they colonize, which guarantees their ability to obtain FAs for energetic and biosynthetic purposes (Nieman et al., 2011). Likewise, in a mouse model of chronic myeloid leukaemia (CML) and in primary human CML samples, a subpopulation of leukaemia stem cells (LSCs), called GAT-LSCs, associates with the gonadal adipose tissue (GAT) upon chemotherapy. GAT-LSCs remain quiescent and secrete pro-inflammatory cytokines, such as IL-1α and TNF-α, to stimulate lipolysis in the nearby GAT adipocytes, which facilitates the uptake and oxidation of free FAs (FFAs) captured by the FA receptor CD36 (cluster of differentiation 36). Interestingly, this metabolic coupling confers GAT-LSCs with the energetic supply to resist chemotherapy and is therefore essential for leukaemia relapse (Ye et al., 2016).

These findings suggest new therapeutic avenues by which to prevent the successful colonization of distant sites by metastatic cells by inhibiting the metabolic crosstalk between cancer cells and their environment. For instance, mouse models of both melanoma (B16) and Lewis lung carcinoma (LLC) have been shown to possess a hyper-glycolytic intra-tumour endothelium that favours metastatic spreading (Cantelmo et al., 2016). Interestingly, the inhibition of the glycolytic activator PFKFB3 (6-phosphofructo-2-kinase) in endothelial cells reduces cancer cell intravasation and metastasis by normalizing tumour vessel architecture (Cantelmo et al., 2016).

We are only just beginning to understand how different local metabolites, and the metabolic programmes they elicit, shape the ability of tumour cells to colonize different sites. In the next section, we discuss recent findings on how specific metabolic cues modulate the behaviour of tumour cells, with an emphasis on the role of FA and lipid metabolism in feeding metastatic progression.

## Fuelling metastatic progression

Recent data suggest that the metabolic profiles of metastatic cells differ as they colonize different organs. For instance, in a murine orthotopic model (Box 1) of metastatic breast cancer, circulating tumour cells (CTCs) purified from blood exhibited increased transcription of the coactivator PPARGC1A (peroxisome proliferator-activated receptor gamma, coactivator 1 alpha; also known as PGC-1α) relative to the primary breast tumour or matched lung metastasis. PGC-1α was shown to determine the metastatic potential of CTCs through enhanced OXPHOS (LeBleu et al., 2014). By contrast, in a mouse model of prostate cancer, PGC-1α was reported to act as a metastasis-suppressing factor, further underscoring the relevance of cancer metabolic heterogeneity within different tumour microenvironments (Torrano et al., 2016).

Another study concluded that breast cancer cells with a broad metastatic potential (bone, lung and liver) could engage both OXPHOS and glycolysis-dependent metabolic strategies, whereas liver metastatic cells relied on glucose uptake and glycolysis, while lung metastatic cells relied on glutamine uptake and OXPHOS to metastasize (Dupuy et al., 2015).

Although these studies provide insights into the dynamic metabolic programmes of tumour cells during cancer progression, further studies will be necessary to mechanistically connect altered gene expression, and the associated bioenergetic behaviour of the cell, to each step of metastatic progression.

Why would metastatic cells that colonize different organs engage in different metabolic preferences? Although tumour cells can hijack nutrients from the cells they encounter as they arrive in an organ, nutrient availability might vary substantially between different organs. For instance, the availability of pyruvate in lungs activates pyruvate carboxylase (PC) in metastatic breast cancer cells, which in turn converts pyruvate to oxaloacetate, thereby enhancing anaplerosis (Box 1) (Christen et al., 2016). Additional studies carried out on *in vitro* three-dimensional (3D) models have revealed that breast cancer cells rely on the non-essential amino acid proline, through proline dehydrogenase enzyme (PRODH) activity, to form spheroids *in vitro* and lung metastases *in vivo* (Elia et al., 2017). Conversely, when breast cancer cells invade the interstitial space of the brain, a region with low glucose concentrations, they enhance gluconeogenesis and the oxidation of glutamine and branched-chain amino acids, which allows them to survive as they extravasate from the brain to blood vasculature (Chen et al., 2015). Cancer cells that metastasize to the brain also use acetate as an alternative substrate for the TCA cycle to support energy and biomass production. This process relies on the activity of ACSS2, which converts acetate to acetyl-CoA and fuels the TCA cycle; notably, *ACSS2* expression is associated with poor survival in patients with gliomas (Mashimo et al., 2014).

Thus, the availability of specific nutrients at different organs might impose unique metabolic states on metastatic cells. However, future studies are required to determine how specific metabolic routes sustain the high energy demand of metastatic cells while modulating the signalling pathways that allow metastatic growth.

### Lipid metabolism in metastasis

Lipid metabolism is often altered in cancer cells. An increased pool of FAs might provide proliferating cancer cells with building blocks for new membranes, signalling metabolites, and substrates for FA oxidation that fulfil the increased energy demand associated with cancer progression. Although FA metabolism has been linked to the growth of primary lesions, including non-small-cell lung cancer and acute myeloid leukaemia (German et al., 2016; Svensson et al., 2016), recent studies suggest that it also associates closely with metastasis. For instance, the internalization of FAs is a main feature of quiescent MICs that eventually promotes their metastasis upon dissemination (Pascual et al., 2017). These MICs are characterized by a high cell-surface expression of the FA receptor, CD36, and have been shown to be solely responsible for initiating metastasis in orthotopic models of human oral squamous cell carcinoma (OSCC), and in experimental metastasis models of human melanoma and of breast cancer (Pascual et al., 2017). Interestingly, human CD36^+^ OSCC metastatic cells in mouse orthotopic models are exquisitely sensitive to circulating blood fat levels, and a high-fat diet or stimulation with palmitic acid strongly boosts these cells' metastatic potential (Pascual et al., 2017). Although the transcriptomic signature of CD36^+^ cells does not strongly associate with EMT, FA uptake by CD36 and by the FA-binding proteins 1 and 4 (FABP1 and FABP4) induces EMT in liver cancer cells, thereby increasing their migration and invasion in *in vitro* assays (Nath et al., 2015).

Besides CD36, the enzyme monoacylglycerol lipase (MAGL), and the FFAs it produces, are elevated in aggressive human ovarian cancer cell lines and in primary ovarian tumours (Nomura et al., 2010). Interestingly, blocking MAGL impairs ovarian tumour growth *in vivo* and ovarian tumour cell migration *in vitro*, and both phenotypes are rescued by exogenous sources of FFAs, including a high-fat diet. These findings further underscore the importance of dietary lipids in promoting malignancy and the migratory capacity of cancer cells (Nomura et al., 2010).

The precise mechanisms by which FAs promote metastasis remain unknown. Louie et al. addressed this question by coupling an isotope-based FA-labelling strategy with metabolomic profiling of different, aggressive human tumour cells, including breast, ovarian, prostate and melanoma (Louie et al., 2013). Their results indicate that these cancer cells use exogenous FAs, such as palmitic acid, as structural lipids and to generate oncogenic signalling (Louie et al., 2013). Other studies, performed in highly aggressive, triple-negative breast cancers (TNBCs; Box 1) indicate that the mitochondrial β-oxidation of FAs meets the high energy demands of metastatic cells as they migrate from the primary tumour to the distant organ (Park et al., 2016). In this sense, acetyl-CoA generated from FA β-oxidation feeds into the TCA cycle to generate large quantities of ATP. Intriguingly, this metabolic pathway seems to be regulated by the proto-oncogenes *Myc* and tyrosine-protein kinase *Src*, two well-known signalling regulators of metastasis (Camarda et al., 2016; Park et al., 2016; Zhang et al., 2009).

Lipid metabolism adaptation by tumour cells has also been demonstrated to play a role in the mechanisms responsible for the adaptation and failure of anti-angiogenic therapies (Sounni et al., 2014). For instance, human and mouse *in vivo* cancer models of breast, colorectal and LLC show a metabolic shift towards *de novo* lipogenesis after therapy withdrawal, which is accompanied by tumour regrowth and a drastic increase in metastatic dissemination. Importantly, the pharmacological inhibition of lipogenesis prevents tumour relapse and metastases (Sounni et al., 2014).

Increased FA uptake and metabolism appears to be a general feature of metastatic cancers, irrespective of the tumour type studied, as shown by the strong correlation that exists between FA uptake and metabolism, and metastatic prevalence and poor patient survival (Nath and Chan, 2016; Pascual et al., 2017). Further studies are required to understand why it is that certain FAs, and their metabolism, associate so closely with metastatic behaviour. Nonetheless, our current knowledge highlights the perils of increased consumption of diets that are rich in added fats in industrialized countries. They also highlight a need for caution concerning ketogenic diets (Box 1), which some believe to be beneficial as an adjuvant cancer therapy. This is because the administration of the ketone compound 3-hydroxy-butyrate in MDA-MB-231 breast cancer xenograft models has been shown to increase and fuel tumour growth (Bonuccelli et al., 2010). Further studies are needed to investigate how diet and lifestyle influence the progression of cancer.

## Metabolism-driven epigenetic alterations in cancer

Cancer is associated with genetic alterations (Curtis et al., 2010; Kreso and Dick, 2014). CSCs of different tumour types carry driver mutations that commonly target stemness-related genes and alter the expression signatures of these cells (Eppert et al., 2011; Merlos-Suárez et al., 2011; Pece et al., 2010). Recent genome-wide sequencing studies indicate that primary lesions and their matched metastases harbour the same set of driver mutations. Since very few cells that harbour these mutations become metastatic, non-genetic alterations must also contribute to the acquisition of metastatic traits (Makohon-Moore et al., 2017; Vogelstein et al., 2013). Indeed, distinct metastasis-associated molecular (transcriptional or proteomic) signatures exist in primary tumours (Nath and Chan, 2016; Pascual et al., 2017; Ramaswamy et al., 2003). In addition, single-cell gene-expression data have recently identified transcriptional signatures that evolve as the metastatic process progresses (Lawson et al., 2015). Thus, although the primary tumour cells harbour the necessary panoply of driver mutations to be metastatic, non-genetic factors must also be required for these cells to exert their full metastatic potential.

### Metabolism shapes the tumour epigenome

Unlike stable genetic traits, epigenetic events are dynamic and reversible, and provide a considerable degree of functional plasticity (Avgustinova and Benitah, 2016). Importantly, large-scale epigenetic modifications have been detected in tumour cells as they colonize distant sites (Bell et al., 2016; Denny et al., 2016; McDonald et al., 2017; Roe et al., 2017). The epigenetic regulation of genes involved in EMT and in mesenchymal to epithelial transition (MET), such as cadherin 1 (*CDH1*), has been extensively documented in a number of malignancies, including breast and hepatocellular carcinomas (Choi et al., 2015; Zhang et al., 2016). Likewise, the acquisition of a neuronal programme that is associated with the metastasis of small-cell lung cancer (SCLC) occurs via the opening up of large-scale chromatin domains and is induced by the transcription factor NFIB (Denny et al., 2016; Minna and Johnson, 2016). Moreover, miRNAs that contribute to the acquisition of metastatic behaviour are controlled by changes in DNA methylation (Mudduluru et al., 2017; Rokavec et al., 2017).

**Fig. 2.**
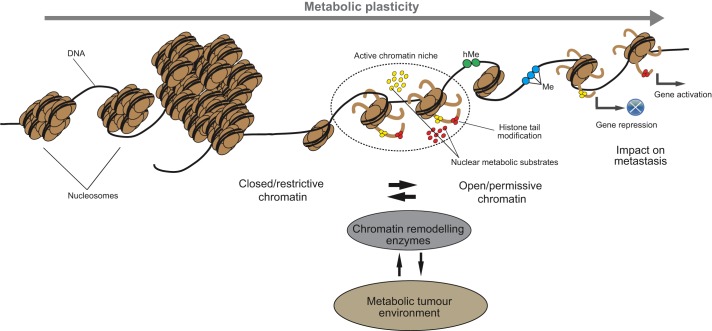
**Epigenetic factors integrate metabolic cues that boost metastatic transcriptional programmes in cancer cells.** Yellow and red circles represent diet-derived metabolites that are utilized by several epigenetic factors in active chromatin niches to post-translationally modify histones and to methylate [blue circles are methyl groups (Me)] and hydroxymethylate [green circles are hydroxymethyl groups (hMe)] DNA. The metabolic status of a cell influences chromatin configuration, via histone tail modifications, to generate transcriptionally restrictive or permissive chromatin. A cell's metabolic status can also influence DNA methylation patterns to regulate gene transcription. This interaction between the cell metabolism and its epigenome can result in unique gene expression signatures that can contribute to the colonization of distant organs and tissues by cancer cells.

Although the molecular mechanisms that underlie chromatin remodelling during metastasis are still largely unexplored, they are influenced by the local and systemic availability of metabolites (Fig. 2). In this sense, several of these metabolites function either as cofactors or substrates for enzymes involved in the deposition of epigenetic marks, thereby directly influencing the epigenetic landscape of tumour cells (Fan et al., 2015). It was recently suggested that enzymes producing some of these metabolic substrates could even colocalize with the epigenetic machinery in the nucleus to generate chromatin metabolic microdomains (Katada et al., 2012). These microdomains would, in turn, facilitate the epigenetic creation of a specific metabolic state in cancer cells.

### Acetyl-CoA: the substrate for histone acetylation

Acetyl-CoA-derived acetyl moieties are transferred to lysine residues on histone tails by histone acetyltransferases (HATs). Histone acetylation leads to the opening up of compact chromatin and to increased transcription of the genes that are located within the acetylated chromatin region. Global transcriptional control through chromatin remodelling in the nucleus has been linked to the enzyme ATP citrate lyase (ACLY) (Wellen et al., 2009). In the cytosol, ACLY converts mitochondria-derived citrate, obtained from glucose oxidation in the TCA cycle, into acetyl-CoA. Importantly, ACLY localizes to the nucleus, where it produces acetyl-CoA that is then used to acetylate the chromatin that encompasses the genes involved in glucose metabolism, such as the glucose transporter 4 (*GLUT4*), *HK2*, *PFK1* and *LDHA* genes. A second enzyme, ACSS2, which is involved in controlling the pool of acetyl-CoA from acetate, translocates to the nucleus, where it can affect the incorporation of acetyl-CoA into histones (Mews et al., 2017). Although ACSS2 can positively or negatively impact tumour progression, depending on the tumour type, its production of acetyl-CoA is required for the growth and progression of liver cancer in murine models (Comerford et al., 2014). Acetyl-CoA generated by ACSS2 is also necessary to fuel breast tumour xenografts, as well as brain metastasis and glioblastoma growth in *in vivo* models (Mashimo et al., 2014; Schug et al., 2015). ACSS2 expression is enhanced in hypoxic and low-lipid environments, providing breast cancer cells with acetate metabolites required for lipid biomass production within the context of metabolic stress (Schug et al., 2015). ACSS2-derived acetate is also essential for energetic purposes because numerous primary and metastatic brain tumours can rely on acetate oxidation for energy, as recently demonstrated in orthotopic models of both human glioblastoma and brain metastasis (Mashimo et al., 2014; Schug et al., 2015). Additionally, the pyruvate dehydrogenase complex (PDC), which normally generates acetyl-CoA from glycolysis-derived pyruvate in the mitochondria, can also translocate to the nucleus to produce acetyl-CoA for histone acetylation at the loci of cell-cycle-progression genes (Sutendra et al., 2014).

Enhancer regions (Box 1) are a focus of current attention since they are highly deregulated in cancer and are the most frequently mutated non-coding regions in human tumours (Abraham et al., 2017; Bal et al., 2017; Bradner et al., 2017; Chapuy et al., 2013; Herz et al., 2014; Mansour et al., 2014; Michailidou et al., 2017; Oldridge et al., 2015; Puente et al., 2015). Interestingly, the acetylation of histone H3K27 at specific enhancers is also required to generate metastatic pancreatic ductal adenocarcinoma (PDA) cells, as seen in a PDA organoid culture system (Roe et al., 2017). The widespread rewiring of enhancers in PDA cells establishes a transcriptional network that aberrantly directs these cells towards embryonic endodermal fate, in a process that is driven by the pioneer transcription factor (Box 1) forkhead box A1 (FOXA1) (Roe et al., 2017).

Lipid metabolism can also contribute to the pool of cellular acetyl-CoA in a cancer-specific manner. When glucose-starved, ACLY-deficient cells are supplemented with FAs (the oxidation of which generates acetyl-CoA in the mitochondria), this supplementation fails to rescue the global loss of histone acetylation (Wellen et al., 2009). However, recent *in vitro* studies in various cancer cell lines have shown that acetyl-CoA obtained by FA oxidation provides acetyl groups that strongly activate a transcriptional programme involved in FA-related processes (McDonnell et al., 2016).

Taken together, these data suggest that multiple carbon sources control the pool of acetyl-CoA to regulate histone acetylation at specific loci in tumour cells. Therefore, acetyl-CoA might be a major force that establishes the transcriptional networks that shape the metabolic flexibility and heterogeneity that cancer cells exhibit as tumours progress.

### Histone methylation

Histone methylation can occur at the arginine (R) and lysine (K) residues of histone 3 (H3) and histone 4 (H4), and is associated with transcriptional activation or repression, depending on the residue targeted and on the number of methyl groups deposited in the N-terminal region of either histone. Histone methylation is tightly regulated by histone methyltransferases (HMTs) and histone demethylases. Similar to DNA methylation (discussed below), alterations in methionine metabolism and in the availability of S-adenosyl methionine (SAM) directly modulate histone methylation. For instance, the levels of histone H3K4me3, a histone mark typically associated with promoter activation, decrease upon *in vitro* methionine restriction in HCT116 cells (Mentch et al., 2015). Importantly, since methionine is an essential amino acid, the activities of HMTs, and of DNA methyltransferases (DNMTs), are strongly influenced by an organism's nutritional status. Indeed, diet-dependent changes in histone methylation have been observed in malignancies such as colorectal adenomas (Pufulete et al., 2005). Significantly, a correlation between enhanced metastatic spread and elevated levels of histone H3 trimethylation has been reported in patient-derived xenograft models of melanoma (Shi et al., 2017).

### DNA methylation

The methylation of CpG islands generally impedes promoter activation and consequent gene expression, although it can also correlate with enhanced transcription factor binding (Domcke et al., 2015; Jones, 2012; Yin et al., 2017). DNA methylation also occurs at actively transcribed gene bodies and at active enhancers (Baubec et al., 2015; Charlet et al., 2016; Dhayalan et al., 2010; Rinaldi et al., 2016). DNMTs are responsible for catalysing the transfer of a methyl group to DNA. However, this modification can be eliminated by the action of ten-eleven translocation (TET) methylcytosine dioxygenases, which initiate the demethylation of DNA by hydroxylating 5-methylcytosines (Scott-Browne et al., 2017).

DNA methylation is vitally important for establishing stable epigenetic states during mammalian development (Rinaldi and Benitah, 2015; Seisenberger et al., 2012; Watanabe et al., 2002). However, in cancer cells, large regions of methylated DNA are hypermethylated, whereas others show widespread hypomethylation (Feinberg and Vogelstein, 1983). In addition, gene body methylation is also altered specifically at genes involved in tumorigenesis (Yan et al., 2015). Although the signals that specifically establish these regions of hyper- and hypomethylation in cancer are not known, it is likely that they are strongly influenced by metabolism. In this sense, the primary methyl group donor for DNA is SAM, which is generated in the methionine cycle from methionine and ATP. After losing the methyl group, SAM is converted to S-adenosylhomocysteine (SAH), which inhibits the activity of DNMTs; SAH can be recycled back to SAM, thus establishing a feedback loop that controls DNA methylation. Furthermore, the TCA cycle intermediate α-ketoglutarate is a co-substrate required for DNA demethylation through TET enzymatic activity, whereas the TCA-cycle-derived metabolites succinate, fumarate and 2-hydroxyglutarate act as competitive TET inhibitors.

How metabolic alterations converge with genetic and epigenetic changes in cancer is unknown. Nonetheless, the activation of the oncogene *KRAS* in pancreatic cancer results in tumours with increased glycolytic flux and serine biosynthesis, in which DNA methylation is fuelled by the increased availability of SAM. This results in the over-activation of DNMTs that control the transcriptional silencing of specific retrotransposons by regulating their methylation status. Hypermethylation of these repetitive elements might promote oncogenic transformation and growth through transcriptional control of host genes (Kottakis et al., 2016). In addition, epidermal stem cells that lack Dnmt3A and 3B upregulate a unique lipid transcriptional network that is associated with CD36^+^ MICs, and are consequently much more predisposed to generating metastatic epidermal tumours (Pascual et al., 2017; Rinaldi et al., 2017). Considering that the genes encoding Dnmt3a and TET proteins are among the most frequently mutated in human cancers (Shlush et al., 2017; Zehir et al., 2017), it will be interesting to study how their mutations influence tumour metabolism, and *vice versa*, during metastatic progression.

Despite the increasing amount of data supporting the importance of metabolism–epigenetics crosstalk in malignancies, we still know very little about the specific impact that metabolism has on altering histone methylation and chromatin architecture in tumour cells. Future studies should aim to investigate how dietary habits affect histone acetylation and methylation in adult stem cells, and how dietary-induced epigenetic changes might predispose adult stem cells to acquire gene expression programmes that can promote metastatic disease.

## Therapeutically targeting metastasis through metabolism

We have reviewed here the growing body of evidence showing that the metabolic plasticity of tumour cells plays an essential role during cancer progression. Tumour cells hijack metabolites from their surroundings and establish different metabolic states to cope with the challenging conditions they face as the tumour grows and colonizes distant sites. Metabolic heterogeneity endows cancer cells with the flexibility required for their growth and metastasis, and could constitute a potential Achilles heel for novel therapeutic strategies to prevent tumour growth and dissemination.

Several therapeutic strategies have already been proven successful in preclinical cancer models (Table 1). For instance, targeting leukotrienes through the inhibitor Zileuton strongly prevents the recruitment of neutrophils, which are required for the formation of a pro-metastatic niche for breast cancer cells in the lung (Wculek and Malanchi, 2015). Targeting lipid metabolism has also produced promising results in preclinical cancer models. For example, in orthotopic models of oral squamous cell carcinoma, blocking the FA receptor CD36 *in vivo* with neutralizing antibodies at early time points of the disease completely prevented metastatic initiation, whereas metastatic regression was only partial when the treatment was initiated at the late stages of the disease (Pascual et al., 2017). In experimental mouse models of melanoma metastasis, the inhibition of *de novo* FA synthesis with Orlistat or thiazolidinediones, or by inhibiting mitochondrial FA β-oxidation with drugs such as etomoxir, a carnitine palmitoyltransferase 1A (CPT1) inhibitor, had anti-tumour effects (Malvi et al., 2015; Nieman et al., 2011; Seguin et al., 2012). Acetyl-CoA synthase (ACS) isoforms catalyse the conversion of long-chain FAs to acetyl-CoA; importantly, several isoforms of ACS show increased expression in different human tumours, including colorectal cancer (Cao et al., 2000). Consequently, the ACS inhibitor triacsin C is under clinical investigation for the treatment of ACS-dependent tumours (Cha and Lee, 2016).

**Table 1.**
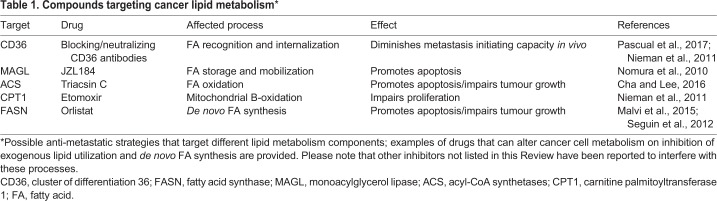
Compounds targeting cancer lipid metabolism*

As mentioned above, the activity of MAGL (which releases FAs from lipid reservoirs) is enhanced in a number of primary tumours, and its inhibition by the drug JZL184 results in reduced pathogenicity in murine models of melanoma and ovarian cancer (Nomura et al., 2010). Interestingly, a high-fat diet prevents the anti-tumour effect of MAGL inhibition, further emphasizing the key role played by exogenous dietary lipids in cancer, and highlighting the potential impact of dietary interventions as additional future therapeutic anti-tumour strategies. As an example of the benefits of dietary intervention, a recent study in both mouse models and humans has shown that, although obesity boosts the metastasis of breast cancer cells to the lung by recruiting neutrophils to the lung pre-metastatic niche, weight loss is sufficient to reverse this effect (Quail et al., 2017).

Finally, the intimate interplay between metabolism and epigenetic mechanisms in cancer opens the possibility of targeting both to obtain synergistic effects (Dawson and Kouzarides, 2012; Rinaldi et al., 2016). The recent development of epigenetic inhibitors, which are already being tested in clinical trials, offers hope in this direction (Avgustinova and Benitah, 2016; Tough et al., 2014).

## Conclusions

Despite intense investigation, the identity of metastatic cells remains elusive, preventing the research community from developing effective anti-metastatic therapies. Several recent studies discussed in this Review indicate that metabolism plays a causal role in the different phenotypic states exhibited by cancer cells during cancer progression and metastasis. Given the plethora of metabolic mechanisms in each organ, it is not surprising that the CSCs that initiate and promote metastasis need to exhibit metabolic plasticity in order to hijack multiple metabolic pathways and to survive hostile microenvironments during the metastatic cascade. Future investigations are needed to characterize and identify the cellular metabolic requirements for initiating metastasis, and to profile the metabolome of pro-metastatic and anti-metastatic niches*.* Such studies should provide clues as to the requirements for treatment and might also identify a window of opportunity for treatment, even leading to disease prevention prior to clinical detection.

The idea that local and systemic factors might confer metastatic cells with enough plasticity to successfully colonize distant organs suggests that influences derived from lifestyles, such as our diet or other daily habits, exert a strong influence on tumour progression, and that such factors could be easily modulated if understood. Increasing data supports the importance of diet in the crosstalk between metabolism and epigenetic regulation (Stefanska et al., 2012), and the extent to which epigenomic changes occur in metastasis (Wong et al., 2017). In the future, it will be necessary to discern which particular components of a diet lead to chromatin changes that are permissive for metastasis. Additionally, the identification of such chromatin modifications and the chromatin-modulating enzymes responsible will point to new potential epigenetic targets for therapeutic intervention.

This article is part of a special subject collection ‘Cancer Metabolism: models, mechanisms and targets’, which was launched in a dedicated issue guest edited by Almut Schulze and Mariia Yuneva. See related articles in this collection at http://dmm.biologists.org/collection/cancermetabolism.
